# Inhibitor of Nicotinamide Phosphoribosyltransferase Sensitizes Glioblastoma Cells to Temozolomide via Activating ROS/JNK Signaling Pathway

**DOI:** 10.1155/2016/1450843

**Published:** 2016-12-20

**Authors:** Jun Feng, Peng-Fei Yan, Hong-yang Zhao, Fang-Cheng Zhang, Wo-Hua Zhao, Min Feng

**Affiliations:** Department of Neurosurgery, Union Hospital, Tongji Medical College, Huazhong University of Science and Technology, No. 1277 Jiefang Avenue, Wuhan 430022, China

## Abstract

Overcoming temozolomide (TMZ) resistance is a great challenge in glioblastoma (GBM) treatment. Nicotinamide phosphoribosyltransferase (NAMPT) is a rate-limiting enzyme in the biosynthesis of nicotinamide adenine dinucleotide and has a crucial role in cancer cell metabolism. In this study, we investigated whether FK866 and CHS828, two specific NAMPT inhibitors, could sensitize GBM cells to TMZ. Low doses of FK866 and CHS828 (5 nM and 10 nM, resp.) alone did not significantly decrease cell viability in U251-MG and T98 GBM cells. However, they significantly increased the antitumor action of TMZ in these cells. In U251-MG cells, administration of NAMPT inhibitors increased the TMZ (100 *μ*M)-induced apoptosis and LDH release from GBM cells. NAMPT inhibitors remarkably enhanced the activities of caspase-1, caspase-3, and caspase-9. Moreover, NAMPT inhibitors increased reactive oxygen species (ROS) production and superoxide anion level but reduced the SOD activity and total antioxidative capacity in GBM cells. Treatment of NAMPT inhibitors increased phosphorylation of c-Jun and JNK. Administration of JNK inhibitor SP600125 or ROS scavenger tocopherol with TMZ and NAMPT inhibitors substantially attenuated the sensitization of NAMPT inhibitor on TMZ antitumor action. Our data indicate a potential value of NAMPT inhibitors in combined use with TMZ for GBM treatment.

## 1. Introduction

Nicotinamide phosphoribosyltransferase (NAMPT) is the rate-limiting enzyme for biosynthesis of nicotinamide adenine dinucleotide (NAD^+^) [1]. NAD^+^ is an essential coenzyme involved in cellular redox reactions. Beside, NAD^+^ has been found to be a critical regulator of many pathogeneses such as obesity, diabetes, nonalcoholic fatty liver disease, and insulin resistance [1–6]. More importantly, NAMPT has a crucial role in cancer cell metabolism [7]. NAMPT expression was found to be increased in numerous types of tumors, including colorectal cancer [8], breast cancer [9], gastric cancer [10], ovarian cancer [11], prostate cancer [12]. In these tumors, inhibition of NAMPT had a profound antitumor effect [8–12]. Thus, inhibition of NAMPT by small-molecule chemical compounds such as FK866, CHS828, and STF-118804 has emerged to be a promising therapeutic strategy for tumor treatment [13]. Although the antitumor action of NAMPT inhibitor has been widely reported, the effect of NAMPT inhibitor on the sensitivity of other anticancer drugs has not been studied extensively.

Glioblastoma multiforme (GBM) is a devastating glioma with no effective treatment yet. GBMs are mainly composed of star-shaped glial cells known as astrocytes [14–20]. Many parameters, such as promotor methylation and micro-RNAs dysfunction, participate in the genesis and development of GBM [21–27]. Previously, radiotherapy is the standard treatment of GBM. In recent years, combined treatment using radiotherapy plus temozolomide (TMZ) becomes the standard treatment for GBM [28]. TMZ is a DNA-alkylating agent that adds a methyl group to the O6 position of guanine in genomic DNA to result in tumor cell death [29]. Compared with radiotherapy alone, TMZ addition increased the median survival from 15.3 months to 21.7 months in patients [30]. Thus, TMZ is a first-line agent with good efficacy and safety in GBM treatment and may increase survival, especially in patients with methylated MGMT promoter [31]. However, high level of MGMT activity in GBM cells would create a resistant phenotype by blunting the therapeutic effect of TMZ [32]. More importantly, little is known on the acquired TMZ resistance.

The specific higher intracellular expression of NAMPT in GBM was discovered by Reddy et al. in 2008 [33]. Their results also showed that serum extracellular NAMPT levels correlated with tumor grade and was highest in GBM [33]. NAMPT inhibitor FK866 induced C6 glioblastoma cell death via inducing autophagic vacuoles, as well as induced nuclei malformation and mitochondria swelling [34]. Recently, Tateishi et al. reported that NAMPT inhibitor conferred metabolic susceptibility in IDH1 mutant gliomas [35]. The NAMPT inhibitor GMX1778 also enhanced the efficacy of radiolabeled somatostatin analog ^177^Lu-DOTATATE treatment and induces a prolonged antitumor response [36].

In the present study, we hypothesized that NAMPT inhibitor may sensitize glioblastoma cells to TMZ-induced cytotoxic effects. We found that FK866 and CHS828, two distinct NAMPT inhibitors, increased the TMZ-induced apoptosis and necrosis, enhanced the TMZ-induced caspase-1, caspase-3, and caspase-9 activities, and augmented the TMZ-induced oxidative stress in glioblastoma cells. In addition, the activated c-Jun/JNK signaling pathway by NAMPT inhibitors may be involved in the sensitizing action to TMZ.

## 2. Materials and Methods

### 2.1. Reagents

Assays for reactive oxygen species (ROS), superoxide anion, and SOD activity were purchased from Cell Biolabs Inc. (San Diego, CA). Assays for measuring caspase-1, caspase-3, and caspase-9 activities were purchased from Beyotime Institute of Biotechnology (Jiangsu, China). Primary antibodies against phospho-JNK, total-JNK, phospho-c-Jun, and total-c-Jun were purchased from Cell Signaling Biotechnology (Danvers, MA). Antibodies against tubulin were purchased from Abcam (Cambridge, UK). The nonradioactive cell viability assay (Cell Counting Kit-8) was purchased from Dojindo Molecular Technologies, Inc. (Kumamoto, Japan). Terminal deoxynucleotidyl transferase dUTP nick end labeling (TUNEL) kit and LDH leakage assay were purchased from Promega (Madison, WI).

### 2.2. Cell Culture

Human GBM cell lines (U251-MG and T98) were purchased from Cell Bank of Institute of Biochemistry and Cell Biology, Shanghai Institutes for Biological Sciences. Briefly, cells were cultured with Dulbecco's Modified Eagle's medium (DMEM) supplemented with 10% fetal bovine serum in an incubator with 95% O_2_ and 5% CO_2_ as described previously [37].

### 2.3. Cell Viability Assay

Cell viability was evaluated by a nonradioactive CCK-8 kit as described previously [37, 38]. U251-MG and T98 cells (5 × 10^3^) were cultured in 6-well plates and treated with TMZ alone (25, 50, 100, 200, and 400 *μ*M), TMZ (25, 50, 100, 200, and 400 *μ*M) plus FK866 (5 nM and 100 nM), and TMZ plus CHS828 (10 nM and 200 nM). At 24 hours after drug treatment, 10 *μ*L of CCK-8 solution was added to the culture medium for 1 h at 37°C. The absorbing at 450 nm was recorded by a microplate reader and the relative cell viability was calculated.

### 2.4. TUNEL Assay

Fluorescent TUNEL staining in glioma cells was conducted as described previously [37]. After 24-hour treatment with different drugs, the culture medium was removed. Cells were incubated in TUNEL reaction solution for 2 hours in the dark. Cells were washed with ice-cold PBS solution gently for three times. Then, cells were incubated with DAPI counterstaining solution for 5 min. The DAPI counterstaining solution was discarded and cells were washed by PBS for additional three times. To examine apoptosis, the stained cells were imaged with a fluorescence microscope (IX81, Olympus). The TUNEL-positive cells were thought to be apoptotic cells (green). The total cell number was evaluated by DAPI staining (blue). At least ten visual fields were counted to calculate the proportion of apoptotic cells.

### 2.5. Lactate Dehydrogenase (LDH) Assay

Cell necrosis was determined with LDH release using CytoTox-ONE LDH leakage assay as described previously [37]. In brief, the cell culture medium at different time points was transferred to a black fluorescence plate and incubated for 10 minutes with CytoTox-ONE reagent followed by stop solution. Fluorescence was measured at 560 nm.

### 2.6. Caspase Activity and Oxidant Stress

The activities of caspase-1, caspase-3, and caspase-9 were evaluated with commercial kits according to the manufacturer's instructions. Caspase-1 activity was measured using a specific tetrapeptide substrate Ac-YVAD-pNA. Caspase-3 activity was measured using a specific tetrapeptide substrate Ac-DEVD-*p*NA. Caspase-9 activity was measured using a specific tetrapeptide substrate Ac-LEHD-*p*NA. In these reactions,* p*NA was generated. The activities of caspase-1, caspase-3, and caspase-9 were quantified by colorimetric detection of free* p*NA at 405 nm.

Four parameters of oxidant stress, including ROS, superoxide anion, total SOD activity, and total antioxidant capacity, were measured using commercial kits from Cell Biolabs Inc. (San Diego, CA) according to the manufacturer's instructions as described previously [6, 37].

### 2.7. Immunoblotting

Immunoblotting assays for phosphorylation of JNK and c-Jun were performed as described previously [37]. Cells were lysed by lysis buffer (50 mM Tris, 150 mM NaCl, 1 mM EDTA, 1% Triton X, and pH 7.4) with protease inhibitors (Pierce, Rockford, IL). After being centrifuged at 12,000*g* for 15 minutes, the supernatant was collected and boiled in 100°C with 2x SDS-PAGE sample loading buffer. Then, the protein samples were run in 10% SDS-PAGE gel (polyacrylamide 12%; 100 V and 30 mA). Gels were transferred onto PVDF membranes and processed for immunoblotting with primary antibodies (MGMT, 1 : 1000; p-JNK, 1 : 500; JNK, 1 : 2000; p-c-Jun, 1 : 500; c-Jun, 1 : 2000; tubulin, 1 : 1000) and by corresponding IRDye-labeled secondary antibodies. Blots were scanned on Odyssey infrared imaging system (Li-Cor, Lincoln, NE).

### 2.8. Statistical Analysis

Data are expressed as mean ± SEM. Differences were evaluated and comparisons between groups were performed by Student's *t*-test or one-way ANOVA with SPSS software (SPSS, Chicago, Illinois). Statistical significance was set at *P* < 0.05.

## 3. Results

### 3.1. NAMPT Inhibitor Sensitizes Glioblastoma Cells to TMZ Treatment

**Figure 1:**
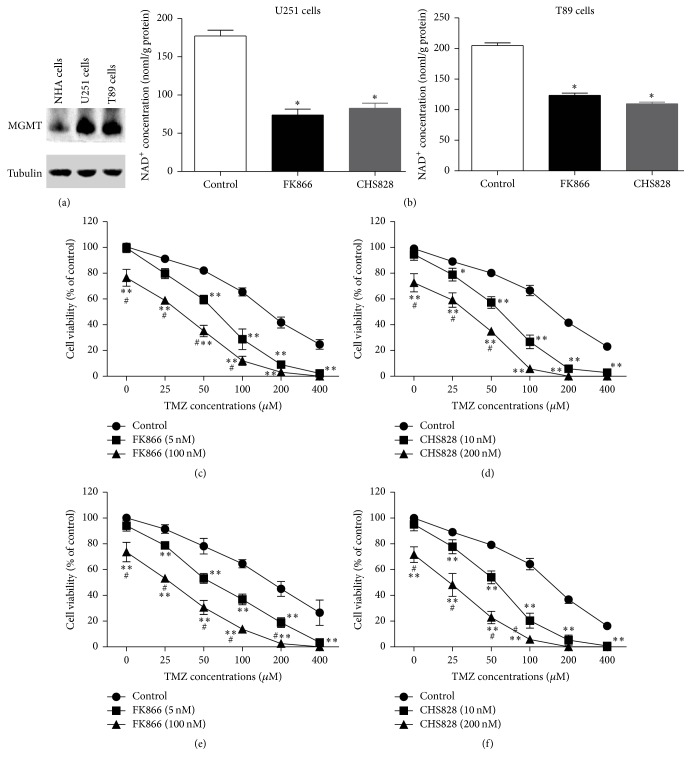
*NAMPT inhibitor sensitizes glioblastoma cells to TMZ treatment*. (a) Upregulation of MGMT in U251-MG and T89-MG cells compared with normal human astrocyte (NHA) cells. (b) Confirmation of inhibitory effect of FK866 and CHS828 on NAD concentration in U251-MG and T89-MG cells. (c) Effects of low (5 nM) and high (100 nM) doses of FK866 on cell viability in U251-MG GBM cells. ^*∗∗*^ *P* < 0.01 versus control. ^#^ *P* < 0.05 versus FK866 (5 nM). *N* = 8. (d) Effects of low (10 nM) and high (200 nM) doses of CHS828 on cell viability in U251-MG GBM cells. ^*∗*^ *P* < 0.05 versus control. ^#^ *P* < 0.05 versus CHS828 (10 nM). *N* = 8. (e) Effects of low (5 nM) and high (100 nM) doses of FK866 on cell viability in T89 GBM cells. ^#^ *P* < 0.05 versus FK866 (5 nM). *N* = 8. (f) Effects of low (10 nM) and high (200 nM) doses of CHS828 on cell viability in T89 GBM cells.

At first, we confirmed the inhibitory effect of NAMPT inhibitor on NAD levels in two human GBM cell lines (U251-MG and T98). MGMT expression was significantly higher in these two cells compared with normal human astrocyte (NHA) cells (Figure 1(a)). As shown in Figure 1(b), both FK866 (5 nM) and CHS828 (10 nM) significantly reduced intracellular NAD levels by ~55–60%. In T98 cells, FK866 (5 nM) and CHS828 (10 nM) inhibited the NAD level by 40–45% (Figure 1(b)). In U251-MG cells, these two inhibitors alone did not significantly decrease cell viability (Figures 1(c)-1(d)). When the doses of FK866 and CHS828 increased to 100 and 200 nM, respectively, the cell viability of U251-MG glioblastoma cells was reduced by FK866 or CHS828 alone (Figures 1(c)-1(d)). In T89 cells, we observed similar phenotypes (Figures 1(e)-1(f)). These data suggest that the low doses of FK866 (5 nM) and CHS828 (10 nM) were noncytotoxic.

In the following experiments, we tested whether NAD^+^ depletion would modulate the sensitivity of TMZ in glioma cells using FK866 at 5 nM and CHS828 at 10 nM. Interestingly, administration of FK866 (5 nM) or CHS828 (10 nM) significantly increased the antitumor action of TMZ in U251-MG and T89 cells (Figures 1(b)–1(f)). Obviously, the combined use of TMZ (25~400 *μ*M) with cytotoxic doses of FK866/CHS828 (100 nM and 200 nM, resp.) resulted in more remarkable antitumor effects in these two glioma cell lines (Figures 1(b)–1(f)). All these results indicate that NAMPT inhibitor sensitizes glioblastoma cells to TMZ.

### 3.2. NAMPT Inhibitor Increases the TMZ-Induced Apoptosis and Necrosis in Glioblastoma Cells

**Figure 2:**
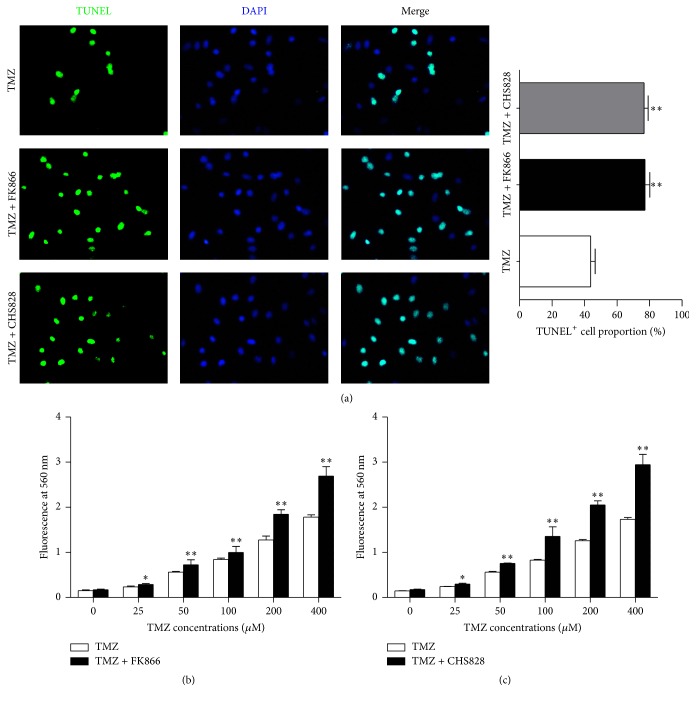
*NAMPT inhibitor increases the TMZ-induced apoptosis and necrosis in glioblastoma cells*. (a) Fluorescent images of TUNEL assay and quantitative analysis showing the effect of low doses of FK866 and CHS828 (10 nM and 20 nM, resp.) on the apoptosis in U251-MG GBM cells. DAPI was used to stain nuclei. ^*∗*^ *P* < 0.05 versus TMZ alone. *N* = 8. At least 20 visual fields were included for analysis. (b-c) LDH assay showing the LDH content in culture medium of TMZ alone, TMZ plus FK866, and TMZ plus CHS828 treated U251-MG GBM cells. ^*∗*^ *P* < 0.05 and ^*∗∗*^ *P* < 0.01 versus TMZ alone. *N* = 8.

We next measured the effects of NAMPT inhibitor on TMZ-induced apoptosis and necrosis using TUNEL assay and LDH release assay, respectively. In TMZ (100 *μ*M) alone treated U251-MG cells, the apoptotic proportion was about ~45% (Figure 2(a)). In the cell treated with TMZ (100 *μ*M) + FK866 (5 nM) or TMZ (100 *μ*M) + CHS828 (10 nM), the apoptotic proportion increased to ~75–80% (Figure 2(a)). LDH assay showed that the LDH contents in culture medium of TMZ + FK866- or TMZ + CHS828-treated cells were higher than those in TMZ-treated cells (Figures 2(b)-2(c)). These results suggest that NAMPT inhibitor is able to increase TMZ-induced apoptosis and necrosis in glioblastoma cells.

### 3.3. NAMPT Inhibitor Enhances the TMZ-Induced Caspase-1, Caspase-3, and Caspase-9 Activities in Glioblastoma Cells

**Figure 3:**
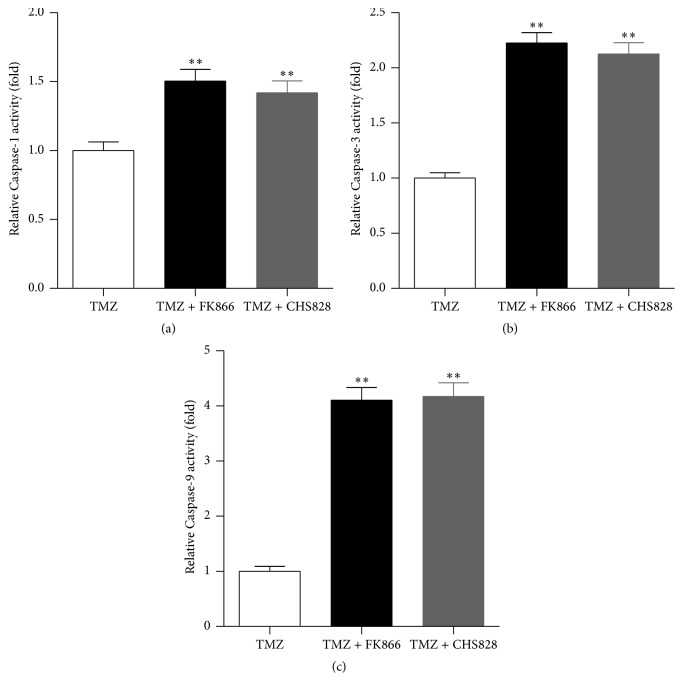
*NAMPT inhibitor enhances the caspase-1, caspase-3, and caspase-9 activities in glioblastoma cells*. (a–c) Effects of NAMPT inhibitors FK866 and CHS828 on the activities of caspase-1, caspase-3, and caspase-9 in U251-MG GMB cells. ^*∗∗*^ *P* < 0.01 versus TMZ alone. *N* = 8.

We compared the activities of caspase-1, caspase-3, and caspase-9 between TMZ and TMZ + FK866 or TMZ + CHS828 in U251-MG cells. As shown in Figure 3(a), FK866 or CHS828 enhanced the caspase-1 activity by ~50%. The activity of caspase-3 was also increased by ~100–120% by FK866 or CHS828 (Figure 3(b)), while the activity of caspase-9 was increased ~3-fold by FK866 or CHS828 (Figure 3(c)). These results suggest that NAMPT inhibitor enhances TMZ-induced caspase-1, caspase-3, and caspase-9 activities in glioblastoma cells.

### 3.4. NAMPT Inhibitor Augments the TMZ-Induced Oxidative Stress in Glioblastoma Cells

**Figure 4:**
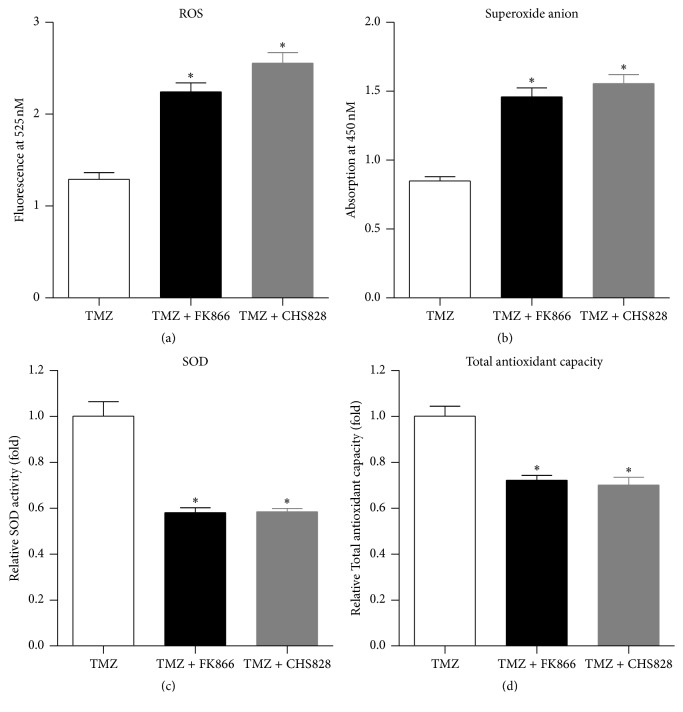
*NAMPT inhibitor augments the TMZ-induced ROS production in glioblastoma cells*. Relative ROS level (a), superoxide anion level (b), total SOD activity (c), and total-oxidant capacity (d) in U251-MG glioma cells. ^*∗*^ *P* < 0.05 versus TMZ alone. *N* = 8.

Acquisition of chemoresistance in gliomas is associated with decreased oxidative stress [39]. Thus, we assessed the effect of NAMPT inhibitor on the TMZ-induced oxidative stress in glioblastoma cells. We found that FK866 or CHS828 significantly increased the TMZ-induced ROS content (Figure 4(a)) and superoxide anion level (Figure 4(b)) in U251-MG cells. Conversely, FK866 or CHS828 reduced the SOD activity (Figure 4(c)) and total antioxidative capacity (Figure 4(d)) in U251-MG glioblastoma cells.

### 3.5. NAMPT Inhibitor Activates JNK Signaling Pathway in Glioblastoma Cells

**Figure 5:**
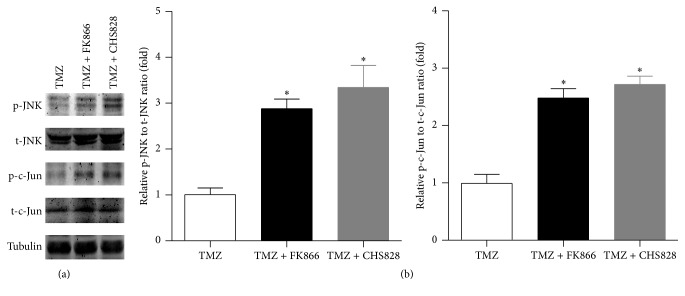
*NAMPT inhibitor activates c-Jun/JNK signaling pathway in glioblastoma cells*. (a) Representative images of phosphorylation of c-Jun and JNK in TMZ alone, TMZ plus FK866, and TMZ plus CHS828 treated U251-MG GBM cells. (b-c) Quantitative analysis on the phosphorylation of c-Jun and JNK in TMZ alone, TMZ plus FK866, and TMZ plus CHS828 treated U251-MG GBM cells. ^*∗*^ *P* < 0.05 versus TMZ alone. *N* = 5.

The c-Jun/JNK signaling pathway functions in a cell context-specific and cell type-specific manner to integrate signals that affect proliferation, differentiation, survival, and migration in tumor [40]. Immunoblotting assay demonstrated that the levels of phosphorylated JNK and c-Jun in TMZ + FK866- or TMZ + CHS828-treated U251-MG glioblastoma cells were enhanced by ~2-fold compared with that in TMZ-treated cells (Figures 5(a)–5(c)). These results suggest that NAMPT inhibitor activated JNK signaling pathway in glioblastoma cells.

### 3.6. JNK Pathway Inhibitor or ROS Scavenger Attenuates the Sensitization of NAMPT Inhibitor on TMZ Antitumor Action in Glioblastoma Cells

**Figure 6:**
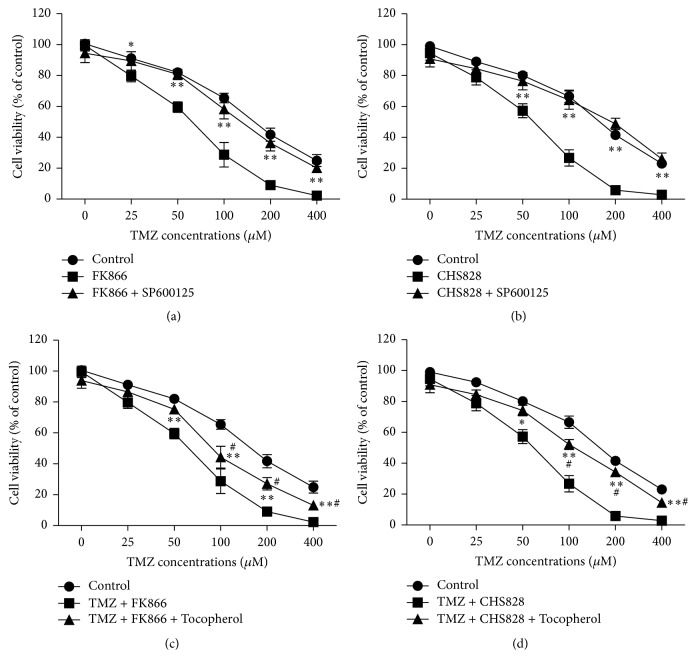
*The c-Jun/JNK pathway and ROS production contribute to the sensitization of NAMPT inhibitor on TMZ antitumor action in glioblastoma cells*. (a-b) Effects of SP600125, a specific inhibitor of JNK pathway, on the antitumor action of FK866 (a) and CHS828 (b). ^*∗*^ *P* < 0.05 versus FK866 (5 nM) or CHS828 (10 nM). *N* = 8. (c-d) Effects of tocopherol, a ROS scavenger, on the antitumor action of FK866 (c) and CHS828 (d). ^*∗*^ *P* < 0.05 versus FK866 (5 nM) or CHS828 (10 nM). *N* = 8. ^#^ *P* < 0.05 versus control. ^*∗∗*^ *P* < 0.01 versus FK866 (5 nM) or CHS828 (10 nM) alone.

Finally we examined effects of c-Jun/JNK pathway inhibitor or ROS scavenger on the sensitization of NAMPT inhibitor on TMZ antitumor action in glioblastoma cells. As shown in Figures 6(a)-6(b), blockade of JNK signaling pathway by SP600125 treatment almost totally abolished the sensitization of NAMPT inhibitor on TMZ antitumor action in U251-MG cells. Moreover, tocopherol, a ROS scavenger, attenuated the sensitization of NAMPT inhibitor on TMZ antitumor action in U251-MG cells (Figures 6(c)-6(d)). These data indicate that both c-Jun/JNK pathway and oxidative stress are required for the antitumor action of TMZ in glioblastoma cells.

## 4. Discussion

In the present study, we showed the first evidence for the sensitizing action of chemical inhibitors of NAMPT to TMZ treatment in GBM cells. The main findings of this study were as follows: (1) administration of low doses FK866 and CHS828 (5 nM and 10 nM, resp.) did not exhibit obvious antitumor action but significantly increased the antitumor action of TMZ in cultured U251-MG and T89 cells; (2) the NAMPT inhibitors increased the apoptotic proportion of tumor cells from ~45% (100 *μ*M TMZ alone) to ~75–80% and enhanced the LDH release from cells, which suggested that the cell death was prompted by the supplement of NAMPT inhibitors compared with a single TMZ treatment; (3) the activities of caspases family and the ROS production, which referred the intracellular proapoptotic activity, were further enhanced by NAMPT inhibitors; (4) NAMPT inhibitors activated c-Jun/JNK signaling pathway in U251-MG and T89 cells; and (5) both blockade of JNK signaling pathway by SP600125 treatment and scavenging intracellular ROS by administration of tocopherol successfully reduced the sensitizing effect of NAMPT inhibitors to TMZ in GBM cells. Taken together, these results demonstrate that inhibition of NAMPT sensitizes glioblastoma cells to TMZ via activating ROS/JNK signaling pathway.

The interest in NAMPT as an antitumor target comes from the discovery on the involvement and significance of NAMPT as a rate-limiting and most crucial NAD^+^ biosynthetic enzyme in mammal cells [41]. As tumor cells always have very high intracellular NAD turnover, which is supportive for the increased metabolic demands in rapid proliferation by generating more ATP/energy [42], NAMPT overexpression is rather common in many types of tumor, including leukemia [43, 44] and solid tumor [8–12]. Indeed, Zoppoli et al. reported that NAMPT inhibitor FK866 resulted in autophagic cell death in primary B-cell chronic lymphocytic leukemia cells via inducing NAD^+^ depletion, mitochondrial transmembrane potential (ΔΨ_m_) dissipation, and ATP shortage [44]. When FK866 was used with tumor necrosis factor-related apoptosis-inducing ligand in leukemia cells, there was a synergistical action in their toxicity towards leukemia cells [44]. Cagnetta et al. confirmed that combined use of FK866 and p-glycoprotein-1 inhibitors showed a strong synergistic cooperative suppression of acute myelogenous leukemia (AML) and B-cell chronic lymphocytic leukemia (B-CLL) sample [43]. In solid tumor, including tumors derived from kidney [45], pancreas [46], lung [47], and breast [48], NAMPT inhibitors have displayed portent anticancer effect.

NAMPT is an important regulator in central nerve system (CNS) biology and CNS disease. A large number of studies have demonstrated that NAMPT participates in the pathophysiological changes of Alzheimer's disease [49, 50], Parkinson's disease [51], cognitive function [52], spinal cord injury [53, 54], axons degeneration [55, 56], and cerebral ischemic stroke [57–61]. So far, the underlying molecular mechanisms of the action of NAMTP in CNS are not fully understood. It has been speculated most of the functions of NAMPT are attributed to its NAD biosynthesis activity. Specifically, NAMPT protein expression was upregulated in brain malignant [62, 63]. Thus, NAMPT was thought to be a good target for GBM treatment. In our study, we found that NAMPT inhibitor FK866 and CHS828 at very low concentration (5 nM and 10 nM, resp.) substantially sensitized two human GBM cells to TMZ treatment. It should be noted that NAMPT inhibitor FK866 (5 nM) or CHS828 (10 nM) alone did not significantly decrease cell viability in our study. Previously, Zhang et al. have showed that FK866 could decrease cell viability of three human-derived glioblastoma cells with IC50 about 25 nM [63]. Similarly, the IC50 of CHS828 in HepG2 hepatocellular carcinoma cells is ~25 nM [42]. In line with these results, our data suggest that although FK866 and CHS828 at very low concentration (5 nM and 10 nM, resp.) are unable to directly induce cell death in GBM cells, they can cause some intracellular dysfunction in these cells which would render the GBM cells more sensitive to TMZ.

In the following mechanistic analyses, we found that FK866 and CHS828 remarkably enhanced ROS production and activated JNK signaling pathway in GBM cells, because the acquisition of chemoresistance in gliomas to TMZ was shown to be associated with decreased ROS production [39]. Recently, Liu et al. demonstrated that evodiamine, a plant alkaloid, induces JNK-mediated autophagy and calcium/mitochondria-mediated apoptosis in human glioblastoma cells [64]. Also, copper compound was found to induce autophagy and apoptosis of glioma cells by reactive oxygen species and activation of JNK signaling pathway [65]. More importantly, JNK activation is a feature of the late proapoptotic response of glioma cells treated with TMZ [66]. Thus, the increases of ROS level and activated JNK activation by FK866 and CHS828 naturally sensitize glioblastoma cells to TMZ. This is the first work to show the activation of JNK signaling pathway by NAMPT inhibitor in tumor cells.

In conclusion, our results demonstrate that specific chemical inhibitors targeting NAMPT sensitize GBM cells to TMZ treatment, evidenced by their synergistic actions on the cell viability, cell death, and apoptotic enzymes activity (caspase family). These phenotypes may be achieved by the enhanced ROS production and activated c-Jun-JNK signaling pathway. These results suggest that NAMPT inhibitors as a sensitizer might be a candidate agent for patients with GBM.
